# The impact of environmental factors in severe psychiatric disorders

**DOI:** 10.3389/fnins.2014.00019

**Published:** 2014-02-11

**Authors:** Andrea Schmitt, Berend Malchow, Alkomiet Hasan, Peter Falkai

**Affiliations:** ^1^Department of Psychiatry and Psychotherapy, LMU MunichMunich, Germany; ^2^Laboratory of Neuroscience (LIM27), Institute of Psychiatry, University of Sao PauloSão Paulo, Brazil

**Keywords:** schizophrenia, affective disorders, environmental factors, neurodevelopment, obstetric complications, animal models, epigenetics

## Abstract

During the last decades, schizophrenia has been regarded as a developmental disorder. The neurodevelopmental hypothesis proposes schizophrenia to be related to genetic and environmental factors leading to abnormal brain development during the pre- or postnatal period. First disease symptoms appear in early adulthood during the synaptic pruning and myelination process. Meta-analyses of structural MRI studies revealing hippocampal volume deficits in first-episode patients and in the longitudinal disease course confirm this hypothesis. Apart from the influence of risk genes in severe psychiatric disorders, environmental factors may also impact brain development during the perinatal period. Several environmental factors such as antenatal maternal virus infections, obstetric complications entailing hypoxia as common factor or stress during neurodevelopment have been identified to play a role in schizophrenia and bipolar disorder, possibly contributing to smaller hippocampal volumes. In major depression, psychosocial stress during the perinatal period or in adulthood is an important trigger. In animal studies, chronic stress or repeated administration of glucocorticoids have been shown to induce degeneration of glucocorticoid-sensitive hippocampal neurons and may contribute to the pathophysiology of affective disorders. Epigenetic mechanisms altering the chromatin structure such as histone acetylation and DNA methylation may mediate effects of environmental factors to transcriptional regulation of specific genes and be a prominent factor in gene-environmental interaction. In animal models, gene-environmental interaction should be investigated more intensely to unravel pathophysiological mechanisms. These findings may lead to new therapeutic strategies influencing epigenetic targets in severe psychiatric disorders.

## Introduction

Schizophrenia is a severe mental disorder starting at young adulthood (Kendler et al., 1996) with a prevalence of about 1% (Jablensky, 1995; McGrath et al., 2008). Each patient suffers from an individual combination of positive, negative, and affective symptoms as well as cognitive deficits, while the severity of these symptoms can change over time depending on the disease stage. Schizophrenia is characterized by prodromal phases with rather unspecific negative and cognitive symptoms, followed by the acute illness with prevailing positive symptoms (Falkai et al., 2011). Remission of psychosis is often incomplete with negative symptoms or even persisting positive symptoms being present in 30% of the sufferers (Hasan et al., 2012) and increasing to 60% in consideration of functionality (Gaebel et al., 2006). Positive symptoms consist of mainly acoustic hallucinations, delusions, disorganized speech, and disorganized behavior as well as thought disorder. The negative symptoms comprise blunted affect, avolition, anhedonia, asociality, and alogia (Crow, 1980; Andreasen et al., 1995). Apart from affective symptoms (e.g., anxiety, depressive mood, and suicidality), another domain refers to cognitive deficits with diminished episodic memory, executive function, and attention (Hoff et al., 1996, 2005; Heinrichs and Zakzanis, 1998; Albus et al., 2002, 2006), which represent a core feature of the disease and are main predictors for poor social-functioning outcome (Green, 1996).

Affective disorders, including major depressive disorder and bipolar disorder with manic episodes, belong to the most prevalent psychiatric diseases. Being among the severe psychiatric diseases (Alsuwaidan et al., 2009), Major depressive disorder has a lifetime prevalence between 16 and 20% (Williams et al., 2007) while lifetime prevalence of bipolar disorder is around 3% in the general population (Merikangas et al., 2007). According to DSM-IV (American Psychiatric Association, 1994) symptoms include loss of energy, social withdrawal, and melancholia in depressive episodes of both major depression and bipolar disorder, and elation, irritability, increased energy with hyperactivity, racing thoughts, pressured rapid speech, decreased need for sleep and an increased involvement of pleasured activities in manic episodes of bipolar disorder. In bipolar disorder, instability of mood is one of the core symptoms, whereas melancholia is the most common sign of depressive episodes (Meyer and Hautzinger, 2003). Furthermore, apart from affective symptoms, both types of affective disorders display impaired cognitive performance, mainly in attention, memory, and executive tasks (Torres et al., 2007). However, because of the individuality of patient's symptoms, current psychiatric diagnostic manuals are not always valid and major psychiatric disorders like schizophrenia, bipolar disorder, and depression are considered as a continuum with different severity of cognitive deficits as common trait (Hill et al., 2013).

**Figure 1:**
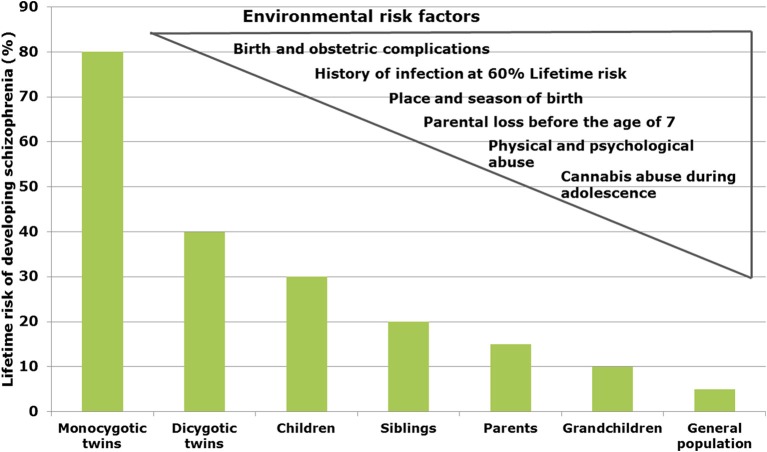
**Interacting risk genes and environmental factors contribute to increase the risk of schizophrenia**. The figure shows the estimated heritability risk to develop schizophrenia as a factor of grade of next of kin. The right side illustrates the contribution of different environmental factors such as infections, obstetric complications, stress periods, and cannabis abuse.

In schizophrenia, twin studies show a heritability of about 60–80% (Sullivan et al., 2003), whereas in bipolar disorder and major depression heritability has been estimated to be 6–80 and 32%, respectively (Wray and Gottesman, 2012). Genome-wide association studies (GWAS) revealed a multitude of genetic risk variants (single nucleotide polymorphisms, SNP) with low effect (Schwab and Wildenauer, 2013). New risk SNPs with high significance are located in genes for Zinc finger protein (ZNF804a), transcription factor 4 (TCF4), micro-RNA 137 (Mir137), the L-type voltage-gated calcium channel (CACNA1C), and CACNB2, Inter-alpha globulin inhibitor H3 and H5 (ITIH3-ITIH4) as well as ankyrin3 (Ank3) with mostly unknown neurobiological consequences (Schwab and Wildenauer, 2013). Ank3 and CACNA1C are also relevant for bipolar disorder (Ferreira et al., 2008) and CACNB2 has been found to be associated with schizophrenia, bipolar disorder and major depression (Cross-Disorder Group of the Psychiatric Genomics Consortium et al., 2013). In a new GWAS study, Ripke et al. (2013) estimated that in schizophrenia about 8.300 SNPs contribute to a common risk of 32%, suggesting that environmental factors interacting with the genetic background contribute to the pathophysiology (Manolio et al., 2009). In schizophrenia, environmental factors are proposed to play a role up to 60% (Benros et al., 2011) (Figure 1).

## Neurodevelopment and psychiatric disorders

During the last decades, schizophrenia has been regarded as a neurodevelopmental disorder. Defective genes and environmental factors may interact to induce symptoms of the disease. The so-called “neurodevelopmental hypothesis” proposes schizophrenia to be related to adverse conditions leading to abnormal brain development during the perinatal period, whereas symptoms of the disease appear in early adulthood after the synaptic pruning process (Weinberger, 1996). In the “two-hit” model, early perinatal insults (genetic background and/or environmental factors) may lead to dysfunction of neuronal circuits and vulnerability to the disease, while a second “hit” during a critical brain development period in adolescence may induce the onset of the disease (Keshavan and Hogarty, 1999). The early perinatal period has been shown to be critical for proper brain development and more specifically the late first and second trimester have been implicated in the pathophysiology of the disease (Fatemi and Folsom, 2009). During adolescence, a synaptic pruning process with excessive elimination of synapses and loss of synaptic plasticity may lead to exacerbation of symptoms in the predisposed brain (Keshavan and Hogarty, 1999; Schmitt et al., 2011a). Additionally, myelination of the heteromodal association cortex like the prefrontal cortex occur during this period (Peters et al., 2012) and decreased fractional anisotropy which corresponds to deficits in myelination has consistently been reported in schizophrenia, suggesting disturbances in fronto-limbic connections (Yao et al., 2013). The prefrontal cortex is highly connected with the hippocampus and this neuronal network has been shown to be disturbed in schizophrenia, mainly due to neurodevelopmental disturbances (Bullmore et al., 1997; Peters et al., 2012; Rapoport et al., 2012). Accordingly, in animal models, perinatal hippocampal lesions induced dysfunction of the prefrontal cortex in adulthood (Lipska, 2004). The disconnection of the hippocampus during brain development alters prefrontal cortical circuitry, function and neurocognition such as prepulse inhibition of acoustic startle response and represents a potent neurodevelopmental animal model for schizophrenia.

Meta-analyses of structural magnetic resonance imaging studies revealed decreased hippocampal volumes and increased ventricles in first-episode schizophrenia patients, confirming the presence of neuropathology before diagnosis is possible (Steen et al., 2006; Vita et al., 2006; Adriano et al., 2012). Even in patients with ultrahigh risk to develop schizophrenia, diminished gray matter of prefrontal and hippocampal regions has been detected compared to healthy controls and in patients experiencing later transition to schizophrenia these volume deficits were yet more pronounced (Witthaus et al., 2009, 2010; Wood et al., 2010). In a comparative analysis, both schizophrenia patients and patients with treatment-resistant major depressive disorder exhibited reduced hippocampal volumes (Maller et al., 2012). Reduced hippocampal volume has also been confirmed in patients with recurrent and chronic depression (Cole et al., 2011). Shape analysis revealed deformations in subfields in the tail of the right hippocampus as well as bilateral volume reductions in patients with first-episode depression (Cole et al., 2010; Meisenzahl et al., 2010), while during the course of the disease further reductions have only been detected in schizophrenia. The presence of alterations in first-episode depression is consistent with a neurodevelopmental hypothesis of early stress experience, especially since the hippocampus plays a major role in inhibiting stress response (McEwen and Magarinos, 2001), providing inhibitory feedback to the hypothalamic-pituitary-adrenal (HPA) axis (Fanselow, 2000).

## Stress during neurodevelopment

Potential stress-inducing factors are migration and urbanicity, which both have been related to schizophrenia. Meta-analyses show an association with urban environment after controlling for minority status (van Os et al., 2010). Individuals living in a higher degree of urbanization had a higher risk to develop schizophrenia than people living in rural areas with a dose-dependent relationship (Pedersen and Mortensen, 2001). In healthy controls, city living was associated with increased amygdala activity, whereas urban upbringing affected the anterior cingulate cortex, affective, and stress response (Lederbogen et al., 2011). In first- and second-generation migrants as well as in minority groups across all cultures, psychotic symptoms have been shown to be increased (Rapoport et al., 2012). According to the “social defeat hypothesis” it has been assumed that social status and degree, e.g., occupying a minority position or experiencing social exclusion, promotes the development of schizophrenia (van Os et al., 2010).

Maltreated children suffer more likely from severe psychiatric disorders such as major depression, bipolar disorder, post-traumatic stress disorder, anxiety disorders, substance abuse and schizophrenia. Childhood maltreatment has been associated with reduced hippocampal volume and amygdala hyperreactivity and also predicts poor treatment outcome (Teicher and Samson, 2013). To date, apart from a genetic vulnerability, stress is widely accepted as risk factor for depression. The stress sensitization hypothesis describes that the first episode of depression sensitizes an individual to stress for which reason subsequent episodes require less stress to be triggered (Shapero et al., 2014). In extension of this hypothesis, early adverse childhood experiences including emotional abuse, physical, and sexual abuse or neglect have been shown to predict depressive symptoms in adulthood (Shapero et al., 2014). Indicating a gene-environment interaction, genetic factors such as polymorphisms in the serotonin transporter or methylenetetrahydrofolate reductase have been reported to interact with developmental stress to increase the risk for depression (Karg et al., 2011; Lok et al., 2013). However, individual genetic background influences the incidence of depression in response to stress and only a part of the persons experiencing stressors develops depression (Keers and Uher, 2012). Moreover, childhood abuse is known to induce psychotic symptoms and suicidal behavior in patients with major depression and bipolar disorder (Arseneault et al., 2011; Tunnard et al., 2014). Early psychotic symptoms represent a risk for developing schizophrenia. In a meta-analysis of 18 case-control studies, Varese et al. (2012) filtered adverse experiences in childhood to significantly increase the risk to develop psychosis and schizophrenia. The neurobiological consequence of stress sensitization involves dysregulation of the hypothalamus-pituitary-adrenal (HPA) axis, contributing to dopamine sensitization in mesolimbic areas and increased stress-induced striatal dopamine release (van Winkel et al., 2008).

The major stress system of the body is the HPA axis, a neuroendocrine system involved in the production of the stress hormone cortisol by adrenal glands. In a subset of patients with major depression, but also in patients with severe psychiatric disorder across phenotypic diagnosis, a dysfunction of the HPA axis has been detected (MacKenzie et al., 2007; Kapur et al., 2012). Depressed patients with a history of childhood abuse have enhanced HPA axis response to psychosocial stress and attenuated cortisol response to application of the synthetic corticosteroid dexamethasone (Heim et al., 2000). In animal models, acute or chronic stress decreased BDNF levels in the hippocampus inclusive the dentate gyrus (Neto et al., 2011). Along with this hypothesis, stress is known to reduce hippocampal dendrites (Magarinos et al., 2011). It additionally increases plasma and adrenal corticosterone levels and application of this hormone reduced hippocampal BDNF levels, mimicking stress reaction (Neto et al., 2011). Chronic stress or repeated administration of glucocorticoids results in degeneration of hippocampal neurons with decreased soma size and atrophy of dendrites (Sapolsky et al., 1990; Watanabe et al., 1992). Thus, volume loss in vulnerable brain regions like the hippocampus as reported for affective disorders may indeed be mediated by stress-induced glucocorticoid neurotoxicity (Arango et al., 2001; Frodl and O'Keane, 2013). In an animal model of depression, the learned helplessness paradigm, inescapable stress induces downregulation of stem cell proliferation (neurogenesis) in the dentate gyrus of the hippocampus (Malberg and Duman, 2003). Stress is additionally known to influence synaptic plasticity in the prefrontal cortex (Rajkowska, 2000). Mediating gene-environmental interactions, epigenetic mechanisms altering chromatin structure such as histone acetylation and DNA methylation may link effects of environmental factors such as stress to transcriptional regulation of specific genes. Depression-like behavior and antidepressant action have been found to be regulated by epigenetic mechanisms (Sun et al., 2013). For example, stress is known to increase histone methylation at the corresponding promoters of the BDNF gene.

Maternal stress during the prenatal period has been related to schizophrenia, depression, and anxiety (Markham and Koenig, 2011), which also applies to autism spectrum disorder and attention deficit hyperactivity disorder (Class et al., 2014). It includes maternal psychological stress exposure e.g., due to bereavement, unwantedness of a pregnancy, natural disaster or war experience (Brown, 2002; Spauwen et al., 2004; Sullivan, 2005; Meli et al., 2012). Children of mothers who experienced e.g., death of relatives or other serious life events developed schizophrenia to a higher degree (Khashan et al., 2008). War experience e.g., during world war II or Israel's Six-Day-War has also been regarded as a critical factor (van Os and Selten, 1998; Malaspina et al., 2008). Especially during the first or second trimester of pregnancy, a vulnerable brain development period may exist for those stress factors. Beside schizophrenia, depression, and anxiety are consequences of exposure to gestational stress (Torrey et al., 1996; Watson et al., 1999; Brown et al., 2000). Prenatal stress is known to influence function of the HPA axis and secretion of glucocorticoid hormones as well protective capacity of the placenta (Owen et al., 2005; Weinstock, 2005, 2008). In addition to effects on stress hormones, prenatal stress influences the fetal transcriptome through microRNA (miRNA) regulation as an epigenetic mechanism, which links environmental factors to altered gene expression in the pathophysiology of schizophrenia and bipolar disorder (Zucchi et al., 2013). Among 435 miRNAs, 19% exhibited reduced expression in the prefrontal cortex in schizophrenia, or more pronounced in bipolar disorder (Moreau et al., 2011). While 18 miRNAs have been found to be differentially expressed, the miRNA miR-497 and miR-29c have been validated to be overexpressed in exosomes of the prefrontal cortex of patients with schizophrenia or bipolar disorder (Banigan et al., 2013). Moreover, methylation or hydroxymethylation of specific genes or promotors regulates gene expression (Akbarian, 2010). Hypermethylation of sex-determining region Y-box 10 (SOX10) has been reported in schizophrenia, whereas in bipolar disorder hypomethylation of HLA complex group 9 (HCG9), ST6 (alpha-N-acetyl-neuranminyl-2,3-beta-galactosyl-1,3)-N-acetylgalactosaminide alpha-2,6-sialyltransferase (ST6GALNAC), and hypermethylation of the serotonin transporter SLCA4 and proline rich membrane anchor 1 (PRIMA1) has been observed (Kato and Iwamoto, 2014). In the frontal cortex of schizophrenia patients, genome-wide methylation analysis revealed differential methylation of 817 genes in promotor regions, among them genes which previously have been associated with schizophrenia (Wockner et al., 2014). Histone modification of chromatin is another epigenetic mechanism to influence gene expression (Peter and Akbarian, 2011). In schizophrenia, altered histone methyltransferases have been detected in the parietal cortex and represent potential future targets for novel treatment strategies (Chase et al., 2013). After prenatal stress in mice, abnormalities in DNA methylation have been described in GABAergic neurons and been related to a schizophrenia-like behavioral phenotype (Matrisciano et al., 2013).

Altered expressions of glucocorticoid receptors and corticotropin-releasing hormone (CRH) in the hippocampus and amygdala have been reported to result from prenatal stress and may be related to increased anxiety and depression-related behavior (Markham and Koenig, 2011). Cognitive deficits of working memory, spatial memory and novel object recognition, related to dysfunctions of the hippocampus and prefrontal cortex, have repeatedly been associated with prenatal stress in animal models and implicate its relationship to severe psychiatric disorders (Markham and Koenig, 2011). Other behavioral consequences are increased locomotor activity and deficits in prepulse inhibition of acoustic startle response (PPI) (Koenig et al., 2005). Increased subcortical and decreased prefrontal dopamine activity after prenatal stress interestingly corresponds to neurotransmitter hypotheses of schizophrenia (Carboni et al., 2010). As a correlate of negative symptoms, social interaction has been reported to be decreased in animals with experience of prenatal stress (Lee et al., 2007). Investigating gene-environmental interaction, a social deficit has been revealed in SNAP-25 knockout mice, which represents a synaptic protein involved in neurotransmitter release, combined with prenatal stress paradigm (Oliver and Davies, 2009).

Some retrospective studies investigated consequences of prenatal food starvation during the “Dutch hunger winter 1944–1945” (Susser et al., 1996; Hoek et al., 1998) and Chinese famine during 1959–1961 (St Clair et al., 2005; Xu et al., 2009). In these investigations, famine episodes of mothers were related to increased risk for schizophrenia in the offspring. Exposure to famine has also been associated with mood disorders and antisocial behavior (Lumey et al., 2011) However, these data are based on ecological inquiries and other factors such as prenatal stress, inflammation, obstetric complications, and toxic substances are not controlled for. Despite these limitations, animal studies revealed effects for protein restriction, choline and vitamin D deficiency on dopamine-related behavior such as locomotor activity and sensorimotor gating, cognition and anxiety, or depression-related behaviors (Markham and Koenig, 2011).

## Birth and obstetric complications

Several meta-analyses have shown an association between complications of pregnancy and delivery and schizophrenia. This applies to obstetric complications of preeclampsia, bleeding, rhesus incompatibility and diabetes, asphyxia, uterine atony, emergency Ceasarian section, and fetal abnormalities such as low birth weight, congenital malformations, and small head circumference. Effect sizes have been estimated between two and three with the highest effect showing emergency Caesarian section, placental abruption, and low birth weight (Cannon et al., 2002a). Schizophrenia has been associated with boys which were small for gestational age at birth (odds ratio 3.2) (Hultman et al., 1999). Children who experienced fetal hypoxia and later developed schizophrenia or affective disorders had basically lower birth weights, indicating that birth weight is a general marker of viability of the intrauterine environment (Fineberg et al., 2013). An odds ratio of 2.0 has been detected by a meta-analysis of Geddes and Lawrie (1995). The same group investigated another meta-analysis of different obstetric complications and found associations between schizophrenia and use of incubator, prematurity and premature rupture of membranes, while low birthweight and use of forceps during delivery were less consistently related to the disorder (Geddes et al., 1999). Maternal bleeding during pregnancy has been found to be associated with schizophrenia with an odds ratio of 3.5 (Hultman et al., 1999). Interestingly, in individuals at high risk for psychosis, those who de facto converted into psychosis had more obstetric complications than non-converting individuals (Mittal et al., 2009). A common factor of all these complications is perinatal hypoxia (Zornberg et al., 2000), which in rats induced a deficit in prepulse inhibition of acoustic startle response (PPI) in adulthood. This behavioral correlate to schizophrenia responded to treatment with the atypical antipsychotic clozapine (Schmitt et al., 2007; Fendt et al., 2008).

The PPI paradigm reflects function of a specific network of brain regions, among them the hippocampus, prefrontal cortex, striatum, and nucleus accumbens (Swerdlow et al., 2001). Especially the hippocampus and basal ganglia are vulnerable to hypoxia-ischemia in the neonate (Morales et al., 2011). Bilateral hippocampal atrophy has been detected in adolescents with a history of perinatal asphyxia diagnosed as hypoxic-ischemic encephalopathy, along with worse verbal long-term memory (Maneru et al., 2003). In schizophrenia, patients with obstetric complications have shown reduced hippocampal volumes (McNeil et al., 2000; Van Erp et al., 2002; Schulze et al., 2003; Ebner et al., 2008), while no effects have been observed in volumes of basal ganglia (Haukvik et al., 2010). However, effects of antipsychotic medication have to be taken into consideration when investigating brain regions (Lieberman et al., 2005). In patients, fetal hypoxia has been related to increased ventricular size and reduced cortical gray matter (McNeil et al., 2000; Cannon et al., 2002b; Falkai et al., 2003), but results are not consistent (Haukvik et al., 2009). Assessment of the two-dimensional gyrification index (GI) revealed no relationship between obstetric complications and cortical folding (Falkai et al., 2007), but after application of a three-dimensional local GI calculation cortical gyrification has been observed to be reduced in the Broca's area in patients and healthy controls with obstetric complication (Haukvik et al., 2012). Since early stages of gyrification take place during gestational week 16 with a rapid increase in the third trimester (Armstrong et al., 1995), this possibly reflects neurodevelopmental disturbances.

In a meta-analysis of 22 studies, the pooled odds ratio for exposure to obstetric complications and subsequent development of bipolar disorder was 1.01 and for development of major depression 0.61, not supporting the association with affective disorder (Scott et al., 2006). However, in a national register study of 1.3 million Swedes, preterm birth has been significantly associated with affective disorders: those with less than 32 weeks gestation had a 2.9-fold higher risk to develop major depression and 7.4% more likely to have bipolar disorder (Nosarti et al., 2012). In a structural MRI study of 79 patients with bipolar disorder and 140 healthy controls from a Norwegion registry, perinatal asphyxia including a hypoxic state lead to smaller amygdala volumes in the bipolar group with perinatal asphyxia, while the non-psychotic group had an association with smaller hippocampal volumes (Haukvik et al., 2013). This is important for the pathophysiology of bipolar disorder, since meta-analyses have revealed smaller amygdala and hippocampus volumes in lithium-naïve patients with bipolar disorder (Hallahan et al., 2011; Hajek et al., 2012).

The consequences of fetal hypoxia comprise neuronal death, white matter damage with impaired myelination and reduced growth of dendrites with more profound effects at mid than late gestation (Rees et al., 2008). Apart from axonal degeneration, especially oligodendrocytes and periventricular white matter are sensible for the influence of oxygen restriction (Kaur et al., 2006). Additionally, an excess of glutamate via hypofunction of the N-methyl-D-aspartate (NMDA) receptor, which has been proposed to play a major role in the pathophysiology of schizophrenia (Hashimoto et al., 2013; Weickert et al., 2013), may damage oligodendroglia and myelin and influence oligodendrocyte differentiation (Mitterauer and Kofler-Westergren, 2011; Cavaliere et al., 2013). Thereby, contributing to cognitive deficits, increased glutamate levels may induce a synaptic imbalance between axons and oligodendroglia, affecting the glial network (syncytium) which is composed of oligodendrocytes and astrocytes (Mitterauer, 2011). In schizophrenia, decreased oligodendrocyte number has been detected in CA4 of the hippocampus and prefrontal cortex (Hof et al., 2003; Schmitt et al., 2009). Although no astrocytosis has been found in schizophrenia (Schmitt et al., 2009), dysfunction of astrocytes may be present in psychiatric disorders (Mitterauer, 2011). At the paranodal junctions between axons and terminal loops of oligodendrocytes, contactin-associated protein is expressed and has been reported to be downregulated in schizophrenia, thus modulating glia-neuronal interaction (Schmitt et al., 2012). In addition to these glial networks, microglia is known to be activated by hypoxic periods and may mediate cell damage via production of nitric oxide synthase, linking neonatal hypoxia to inflammatory processes (Kaur et al., 2006). In the rat model of perinatal hypoxia, cDNA microarray derived analysis revealed synaptic genes like complexin 1, syntaxin 1A, SNAP 25, neuropeptide Y, and neurexin 1 to be deregulated in several cortical regions and striatum during adulthood. In this study, clozapine treatment had effects on gene expression (Sommer et al., 2010). These findings are relevant to schizophrenia, which has been described as a disease of dysconnectivity on the synaptic and systematic level (Schmitt et al., 2011a, 2012).

## Inflammation during pregnancy

As to offspring of mothers exposed to influenza, several epidemiological studies have demonstrated an increased risk for schizophrenia. However, infections with other viruses such as measles, rubella, varicella-zoster, polio, and herpes as well as bacteria and parasites (*Toxoplasma gondii*) also confer an increased risk of schizophrenia (Hagberg et al., 2012). Moreover, maternal infections and subsequent inflammatory processes and brain injury during pregnancy are known to be associated with preterm labor, especially at <30 weeks of gestation (Dammann et al., 2002; Goldenberg et al., 2008). These complications are known to affect white matter structures such as corpus callosum or other major white matter tracts and may be associated with neurodevelopmental injury of oligodendrocytes in schizophrenia (Chew et al., 2013). In fact, decreased numbers of oligodendrocytes have been detected in the hippocampus and prefrontal cortex in post-mortem brains of schizophrenia patients and may affect subsequent myelination (Hof et al., 2002; Schmitt et al., 2009). Pro-inflammatory cytokine release has been described as common mechanism of infectious processes (Brown, 2012; Garbett et al., 2012). In the prefrontal cortex of schizophrenia patients, gene expression analysis revealed increased expression of inflammatory genes along with activation of microglia (Beumer et al., 2012; Fillman et al., 2013), but results in the superior temporal cortex also point to the reduced expression of immune-related genes (Schmitt et al., 2011b). In which manner these post-mortem findings are related to perinatal insults is not yet resolved. In animal studies, maternal infection induced behavioral abnormalities in early adulthood comparable to schizophrenia such as deficits in PPI, social interaction and working memory (Meyer and Feldon, 2009).

## Challenging future investigations: gene-environmental interaction

Many efforts have been made to unravel the genetic background of severe psychiatric disorders. Recent GWAS point toward a partial overlap in susceptibility between schizophrenia and affective disorders (Cross-Disorder Group of the Psychiatric Genomics Consortium et al., 2013). For example, the risk variant of the alpha 1C subunit of the L-type voltage-gated calcium channel (CACNA1C) gene is associated with schizophrenia, bipolar disorder and major depression (Green et al., 2010). This genotype has been shown to influence hippocampal activation during episodic memory encoding and retrieval (Krug et al., 2013). However, effect sizes for common genetic variants so far were small (Brown, 2011; Réthelyi et al., 2013). Environmental factors, especially those affecting molecular and structural processes in relevant brain regions during neurodevelopment, are supposed to interact with genetic factors to induce severe psychiatric disorders (Harrison and Weinberger, 2005). For example, a large number of schizophrenia candidate genes are known to be regulated by hypoxia (Fatemi and Folsom, 2009; Schmidt-Kastner et al., 2012). In transgenic animal models of schizophrenia, stressful events have been induced to reinforce the behavioral phenotype (Haque et al., 2012; Hida et al., 2013). Future animal studies should combine risk variants of susceptibility genes with several environmental factors such as perinatal infection, stress and hypoxia to develop valid models of severe psychiatric disorders. These models could be useful to understand pathophysiological brain mechanisms and to develop new treatment strategies aiming at risk-based therapy and prevention of symptoms of severe psychiatric disorders.

### Conflict of interest statement

Berend Malchow declares no conflicts of interest. Alkomiet Hasan has been invited to scientific conferences by Janssen Cilag, Pfizer, and Lundbeck. He received paid speakership by Desitin and is member of the Roche advisory board. Andrea Schmitt was honorary speaker for TAD Pharma and Roche and has been member of the Roche advisory board. Peter Falkai until 12/2011 has been member of the advisory boards of Janssen-Cilag, BMS, Lundback, Pfizer, Lilly, and AstraZeneca and received an educational grant from AstraZeneca and honoraria as lecturer from Janssen-Cilag, BMS, Lundbeck, Pfizer, Lilly, and AstraZeneca.
